# Environmental Factors that Impact the Workplace Participation of Transition-Aged Young Adults with Brain-Based Disabilities: A Scoping Review

**DOI:** 10.3390/ijerph17072378

**Published:** 2020-03-31

**Authors:** Saeideh Shahin, Meaghan Reitzel, Briano Di Rezze, Sara Ahmed, Dana Anaby

**Affiliations:** 1 School of Physical and Occupational Therapy, McGill University; Montreal, QC H3J1Y5, Canada; sara.ahmed@mcgill.ca (S.A.); dana.anaby@mcgill.ca (D.A.); 2 Centre de Recherche Interdisciplinaire en Réadaptation de Montréal Métropolitain (CRIR); Montreal, QC H3S1M9, Canada; 3 School of Rehabilitation Science, McMaster University; Hamilton, ON L8S1C7, Canada; reitzelm@mcmaster.ca (M.R.); direzzbm@mcmaster.ca (B.D.R.); 4 CanChild Center for Childhood Disability Research; Hamilton, ON L8S1C7, Canada

**Keywords:** young adult, employment, workplace, labor force, environmental impacts, social environment

## Abstract

Workplace participation of individuals with disabilities continues to be a challenge. The International Classification of Functioning, Disability and Health (ICF) places importance on the environment in explaining participation in different life domains, including work. A scoping review was conducted to investigate environmental facilitators and barriers relevant to workplace participation for transition-aged young adults aged 18–35 with brain-based disabilities. Studies published between 1995 and 2018 were screened by two reviewers. Findings were categorized into the ICF’s environmental domains: Products and technology/Natural environment and human-made changes to environment, Support and relationships, Attitudes, and Services, systems and policies. Out of 11,515 articles screened, 31 were retained. All environmental domains of the ICF influenced workplace participation. The majority of the studies (77%) highlighted factors in the Services, systems and policies domain such as inclusive and flexible systems, and well-defined policies exercised at the organizational level. Social support mainly from family, friends, employers and colleagues was reported as a facilitator (68%), followed by physical accessibility and finally, the availability of assistive technology (55%). Attitudes of colleagues and employers were mostly seen as a barrier to workplace participation (48%). Findings can inform the development of guidelines and processes for implementing and reinforcing policies, regulations and support at the organization level.

## 1. Introduction

Participation, defined as “involvement in a life situation” by the International Classification of Functioning, Disability and Health (ICF) [1], is one of the main rehabilitation goals among people with disabilities [2]. Participation in work is particularly important for transition-aged young adults living with a disability which involves transition to many new adulthood roles; however, this group often experiences increased participation limitations over time, in this pertinent life area [3]. 

Generally, employment is associated with improved physical, psychological and social well-being [4]. Having work experience is important for young adults, especially for those with disabilities, as it increases the likelihood of attaining postsecondary employment later in adulthood [5]. Despite its known benefits, young adults with disabilities in North America [6] and around the world have the lowest employment rates, between 30%–53% [7]. This group also experiences higher rates of poverty when compared to those without disabilities [8]. Focusing on this vulnerable transition-aged group is critical as it involves transitioning to adulthood roles and requires support to ensure successful experiences in their early stages of employment. Such support is important since open and competitive employment settings do not always have the knowledge and resources to make appropriate accommodations [9].

Environmental factors, referring to the physical, social, attitudinal and institutional facets of the environment, are known to affect participation outcomes [10]. These factors can either act as facilitators and enhance one’s functioning and participation, and/or serve as barriers impeding one’s engagement in meaningful activities [1]. Hence, the environment may explain some of the discrepancies in employment rates among young adults with disabilities [11,12]. Research suggests that the environment can serve as a promising target for interventions to improve participation. Additionally, in many cases, change at the level of the environment is a more practical target rather than at the level of the individual [10]. Understanding the challenges that the environment poses for participation in the workplace among this population can inform such interventions. Recent knowledge syntheses have illustrated the impact of environmental modifications on workplace participation among adults with autism spectrum disorder (ASD) [13] and workplace culture on the participation of people with intellectual disability (ID) [14]. However, to date, no scoping review has been completed to comprehensively synthesize the knowledge-base related to the environmental effects on the workplace participation among the understudied population of transition-aged young adults with various brain-based disabilities [15].

This scoping review aimed to identify and synthesize the existing evidence on the impact of environment on participation in mainstream inclusive work settings among transition-aged young adults with brain-based disabilities. Brain-based disabilities refer to any neurologically based congenital or acquired conditions, as well as neurologically chronic conditions (e.g., cerebral palsy, brain- and spinal-related injuries) including sensory disorders. Such an initiative will also reveal current gaps in knowledge within the field of employment in brain-based disability, informing future research.

## 2. Materials and Methods 

A scoping review methodology was applied, allowing us to map and broadly cover the breadth of current knowledge regarding the environmental factors that impact employment participation of transition-aged individuals [16]. The 5-stage method for scoping reviews by Arksey and O’Mally [16] and advanced by O’Brien, Colquhoun and Levac [17] was used. 

### 2.1. Identifying the Research Question

Typical to scoping reviews, a broad question was identified as follows: What is known about the impact of the environment on the participation in the work setting among transition-aged individuals with brain-based disabilities? 

### 2.2. Identifying Relevant Studies

A systemic search of studies published between 1995 and June 2018 was conducted. Five relevant databases covering a range of research areas including health, social and rehabilitation sciences were consulted: OVID MEDLINE, EMBASE, PsycINFO, PubMed and CINHAL. The input of an expert librarian ensured that all relevant publications were included. The following search terms (see Table 1) were utilized to capture the multi-faceted aspects of the environment combined with OR: physical environment, social environment, cultural environment, institutional environment, built environment, attitudes, workplace, accessibility, services, policy, social support, and relationships. Comprehensive keywords were used to capture the concept of ‘work participation’, using terms representing ‘participation’ (e.g., engagement, involvement) combined with terms illustrating ‘employment’ (e.g., job, productivity). These three categories of terms were combined with the term ‘brain-based disability’ and related conditions (for further details see Table 1) using AND. Both Medical Subject Headings (MeSH) and keywords were used. Final searches resulted in 14119 articles, which were organized via EndNote reference manager. The removal of duplicates resulted in 11,515 articles. 

### 2.3. Study Selection

Empirical peer-reviewed studies, regardless of their design, were included if they: (1) explored the relationship between the environment and participation in an open competitive workplace, (2) targeted transition-aged young adults between the ages of 18–35 years old (based on the mean) with acquired or congenital brain-based disabilities, and (3) were published in English. This age range was chosen as it reflects a period of transitioning to adulthood, which involves greater independence, acquiring employment, and maintaining relationships and leisure activities [18]. Full-time employment usually begins at 18 [19], and because dependency on family is prolonged within this population, this transition phase was extended to the mid-30s [20]. Articles were excluded if they had the following characteristics: (1) theoretical, conceptual or opinion papers, (2) studies whose participants’ primary diagnosis was a mental health condition, (3) studies that only focused on recommendations to occupational health and safety guidelines in the workplace or included only descriptions of work hardening programs, vocational rehabilitation programs and facility-based programs, or the impact of the environment on these programs. Three researchers independently screened an initial set of 50 articles by title and abstract, attaining a 90% agreement [21]. The remaining articles were equally distributed and screened by title/abstract, resulting in 221 studies retained for full-text screening by two researchers. Any disagreement was resolved through discussions and consultations with the senior investigator. Finally, 25% of the included and excluded articles were randomly selected and validated by a rehabilitation specialist, independent of the study. Consensus was reached through a discussion. 

### 2.4. Extracting and Charting Results

A data extraction sheet containing the reference, year and country of publication, type of study and design, study purpose, number and age of participants, diagnosis, place of employment, aspects of the environment and participation, main findings, and utilized assessment tools was created using Excel. Elo and Kyngäs’ [22] coding and categorization process was used to classify data according to the five environmental domains of the ICF framework: Products & technology (e.g., assistive devices, built environment), Natural environment and human-made changes to environment (e.g., geographic location, climate), Support & relationships (e.g., including family, friends, colleagues, and healthcare professionals), Attitudes (e.g., belief, values and perceptions of others), and Systems, services & policies (e.g., programs, regulations). This comprehensive framework was selected as it accords special attention to the role of the environment on participation [23]. The Products and technology domain was combined with the Natural environment and human-made changes to environment domain into one category as they both relate to the physical environment, resulting in four domains of the environment. Main findings categorized into the ICF environmental domains were jointly validated by two researchers followed by input from the senior researcher [24]. 

### 2.5. Collating, Summarizing and Reporting the Results

A descriptive summary of each article is presented with regards to the following elements (see Table 2): author, year, country, aim of the study, study design, population (number, age, diagnostic), ICF environmental domains included, and summary of the main findings. Data was described in terms of the percentage of the articles that explored specific environmental domains of the ICF. Additionally, findings were synthesized to explore the range of identified environmental barriers/facilitators that contribute to young adults’ workplace participation. A table (see Table 3) summarizing findings in terms of environmental barriers and facilitators per each ICF environmental domain was also created. 

## 3. Results

**Figure 1:**
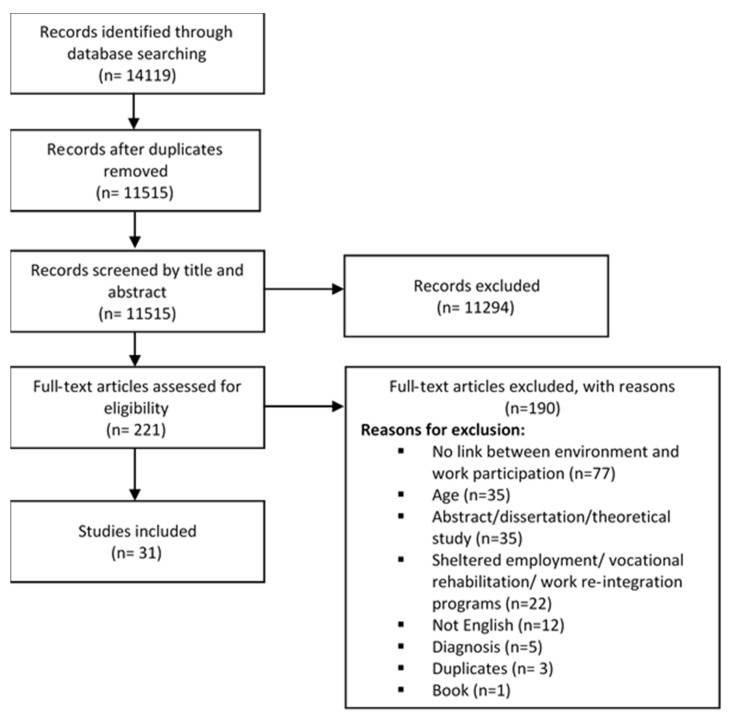
Flow chart of study selection process.

Thirty-one articles met the inclusion criteria (See Figure 1). One hundred and ninety articles were excluded and the reason for exclusion is specified in Figure 1. The validation process, conducted by the rehabilitation specialist, resulted in 100% agreement for included articles and 92% agreement for excluded articles. The initial disagreement on 8% of the excluded articles was resolved, and agreement was reached after a discussion with the senior researcher. 

### 3.1. Descriptive Summary of the Studies 

The selected studies were published between the years of 1995 and 2018 with 74% of the studies (*n =* 23) having been published during or after 2010. The majority of the studies were qualitative (*n =* 17, 55%), followed by quantitative (*n =* 11, 35%), and literature reviews (*n =* 3, 10%). The mean age of the participants was less than 35 years old in 28 of the studies included. The participants in the remaining three studies had a mean age between 35 to 65 years old and were included because data could be extracted specifically to participants aged 35 and younger. Studies were most often completed in the US (*n* = 10), Canada (*n =* 5), Australia (*n =* 4) and the Netherlands (*n =* 3). Single studies from Brazil, China, Namibia, New Zealand, South Africa, Sweden and the UK were also included. Two studies had representation from more than one country. 

Intellectual or developmental disability (*n =* 9), sensory impairments including vision and hearing loss (*n* = 7) and cerebral palsy (CP) (*n =* 6), were the brain-based disabilities most frequently examined in the included studies. Other brain-based disabilities examined include spinal cord injury (SCI) or other spinal conditions, muscular dystrophy (MD), learning disability (LD) or dyslexia, epilepsy, spina bifida (SB), autism spectrum disorder (ASD), multiple sclerosis (MS), attention-deficit hyperactivity disorder (ADHD), traumatic brain injury (TBI) and other neurological conditions. Selected studies included perspectives of young adults (*n =* 28), parent or caregivers (*n =* 5), employers (*n =* 4), health care providers or unspecified support persons (*n =* 2) and vocational support specialists (*n =* 3). Six of the articles reviewed included multiple stakeholder perspectives. 

Many of the qualitative studies (*n =* 17) utilized interviews or focus groups as their primary means of collecting data from participations. Five of the 31 included studies utilizing outcome measures to collect data/information about work participation. These measures included the Assessments of Life Habits [24], the Work Experience Survey [25], the Career Mastery Inventory [25], the Beach Centre Family Quality of Life Scale [26], the Developmental Behaviour Checklist adult version [26], the Index of Social Competence [26], the Stages of Change work Participation Scale [27], and the Vocational Integration Inventory [28]. Only one standardized measure addressed all aspects of the environment; the Measure of the Quality of the Environment [24], while the others focused on a single-domain measure of the environment such as the Family Support questionnaire [26]. Other studies identified environmental factors in the workplace by either relying on data from national surveys or by using their own questionnaires/surveys without any psychometric tests to validate them [27,28,29,30,31,32].

**Figure 2:**
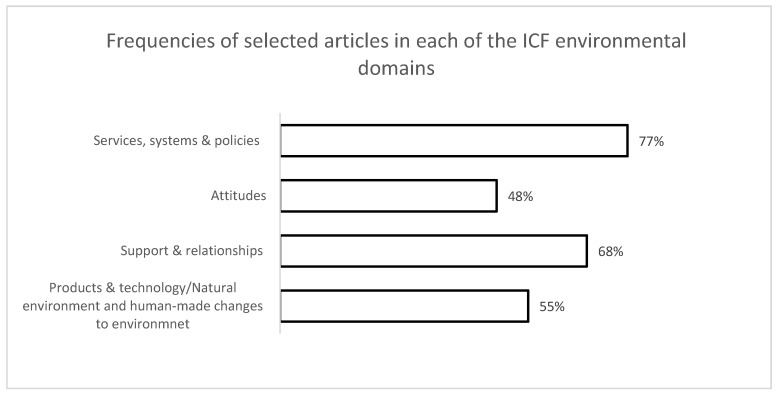
Frequencies of selected articles in each of the International Classification of Functioning, Disability and Health (ICF) environmental domains.

The majority of the included studies (71%) examined more than one facet of the ICF environmental domains with regards to work participation. The domain of Services, systems and policies (*n =* 24, 77%) was most frequently examined in the literature followed by the Support and relationships (*n =* 21, 68%), Products & technology /Natural environment and human-made changes to environment (*n =* 17, 55%), and Attitudes (*n =* 15, 48%) (see Figure 2).

### 3.2. Main Findings

#### 3.2.1. Products and Technology/Natural Environment and Human-Made Changes to Environment

Among the reviewed articles, 17 (55%) addressed the role of the physical and sensory environments on young adults’ participation in the workplace. Identified barriers included the lack of physical accessibility and assistive technology, inflexible and unreliable transportation systems and in some cases, inadequate lighting and temperature of the work setting [30,33,34,35]. To illustrate, participants with osteogenesis imperfecta, spina bifida or other impairments caused by accidents in the US and in Norway, required workplace accommodations related to the built environment (e.g., accessible paths and bathrooms, ramps, railings, door handles), assistive technology (e.g., voice recognition software), and ergonomic office tools (e.g., a specialized mouse or an adjustable desk) to promote their performance and engagement in the workplace [33]. The sensory environment, including lighting and temperature, also influenced the employee’s ability to effectively perform his/her tasks. For example, the brightness of the environment often caused headaches or impeded computer work due to excessive reflection of light on the desktop among employees with TBI [25].

Studies also discussed the consequences associated with physical environment barriers and the perceived cost of adapting the environment. Failure to provide appropriate accommodations resulted in embarrassing situations and prevented persons with a disability to perform their responsibilities to the best of their abilities [30]. The cost of providing accommodations and adapting the physical environment was reported as a barrier to acquiring a job [29]. In fact, young adults reported that requiring fewer physical adaptations in the workplace increased their chance of acquiring a job [32].

Many studies found that access to adequate transportation is imperative for acquiring and retaining employment [34,35,36]. Long distance transportation was depicted as a hindrance to working [29]. In fact, transportation was a significant predictor of paid employment amongst young adults with mobility, hearing, vision, communication and/or cognitive impairments [12,37]. Flexible and timely transportation was found to support employment of those with physical disabilities [33]. Additionally, access to a vehicle as either a passenger or driver increased the likelihood of acquiring employment among young adults with various types of disabilities [37]. Lindsay [37] also reported the impact of geographical location on employment rate for individuals in their early years of transitioning who use mobility devices: those living in urban areas were more likely to find a job compared to those living in rural areas. This finding could be explained by other environmental barriers common in these geographical areas, such as a poor economy, scarcity of jobs and lack of services in certain areas that disadvantage people with disabilities [24,26,37].

Environmental supports were also identified; an accessible work environment in which accommodations were made to meet the employee’s needs, optimized performance and facilitated engagement in the workplace [30,38]. Many employees reported working from home [25,33,38] and using assistive technology such as Dictaphones, dual monitors, assistive devices for communication and computerized phones and alarms, positively impacted work satisfaction and work maintenance [33,39,40,41].

#### 3.2.2. Support and Relationships

Twenty-one articles (68%) fell under this category. The main barriers involved young adults’ lack of social support or their perception of low support from parents [38]. However, interestingly, those with autism [42] and spina bifida [34] who had high parental support or overprotective parents were even less likely to be employed. Hence, family members, especially parents, played a significant role in finding and maintaining employment [31,43]. The main barriers to employment opportunities for those with autism [44] and intellectual disabilities [26,45] included lack of parental support, time, awareness and knowledge of abilities, parental fatigue and unwillingness to facilitate job search. Family involvement facilitated finding and maintaining employment by guiding career planning and adequate job search, providing support at the workplace, and in some cases, assisting with transportation [27,44,45]. Additionally, having parents with high work-related expectations, who advocated supported employment and provided emotional support, increased the likelihood of being employed and meeting the demands of the job on a daily basis [45] among those with learning disabilities [30] and various types of disabilities [46].

Additional social support from peers and co-workers also emerged as a main facilitator for employment. Sung and Connor [27] demonstrated that in the presence of other important factors (e.g., self-efficacy), 22.5% of the variation in employment among transition-aged individuals with epilepsy was explained by the support they received from parents, friends and professionals. This involved helping them develop specific independence skills required in the workplace [27,34]. Peer support, especially from those already employed, was another facilitator that encouraged and motivated individuals with brain-based disabilities to look for employment [47]. In addition, engagement in work was facilitated in inclusive workplaces in which interaction between co-workers was encouraged [32,44]. In fact, some of the strategies that service agencies used to support the integration of young adults with disabilities included building relationships and prompting co-workers and supervisors to actively invite employees to socialize during breaks, lunches and while performing the job [44]. Furthermore, a systematic review by De Beer et al. [38] indicated that assistance from colleagues was among the supports that facilitated employment for young adults with developmental dyslexia. To illustrate, having colleagues proofread their work predicted better employment outcomes [30,35], and positive interactions in the workplace led to their career advancement [46]. Participating in work-related social activities such as going to staff functions, eating lunch with other employees and developing interpersonal relationships with co-workers that expanded beyond the workplace, also increased the likelihood of employees with intellectual disabilities to keep their job [28,48].

Management styles within the organization played a role in work experiences of this transitioning population. Approachable managers who created inclusive and fair work environments, as well as those who built relationships and created a strong sense of teamwork, increased engagement in the workplace for those with developmental disabilities [28]. Similarly, managers who had direct contact with their employees, closely collaborated with employment service providers and allowed for work trials rather than interviews, facilitated the employment of young adults with ASD [49]. Moreover, young adults with disabilities were happier in workplaces where they were treated equally [33] and felt that their skills and opinions were valued by the managers [49].

#### 3.2.3. Attitudes

This environmental factor was addressed in 15 (48%) studies in which attitudes of others towards persons with a brain-based disability was mainly seen as a barrier to their employment and participation in the workplace. Young adults with a disability often experienced prejudice and stigma from their employers and co-workers in the workplace. For example, they generally got hired for less skilled occupations as their employers did not believe in their abilities [30,32]. Lindsay et al. [47], illustrated the misconceptions from employers regarding the functional abilities of people with physical disabilities and the negative impact of societal attitudes on their employment. Additionally, many young adults with brain-based disabilities hesitated to disclose their diagnosis (e.g., learning disabilities) to their employer due to fear of discrimination [30]. In their systematic review, De Beer et al. [38] revealed that the reaction of co-workers to this transition-aged population was mostly negative. This negative attitude which usually stems from a lack of knowledge, led to negative experiences for the employee when seeking out a job, i.e., increased stress during the interview, as well as in retaining a position [34,50,51]. In other words, this prejudice created obstacles in young adults’ abilities to acquire and enter the labor market or to advance in their careers [30,52]. For example, stereotypes associated with this population such as their inability to work, their need for costly accommodations or their unwillingness to be active members, hindered persons with a disability to exhibit and exercise their skills in the workplace. This was evident in various types of brain-based disabilities, including physical, intellectual and sensory related impairments [39,52,53]. In one study, it was found that this negative perception and discrimination led to higher rates of unlawful discharge of young adults with epilepsy as compared to their colleagues [51]. Overall, approachable employers with positive attitudes and sensitivity to the needs of the employee created positive work experiences and led to better employment satisfaction [30,49,50].

#### 3.2.4. Services, Systems and Policies

The majority of the studies (*n =* 24, 77%) focused on the impact of services, systems and policies on both acquiring/finding a job and maintaining participation in the workplace. Internal factors, those within the organization/workplace, and external factors, those outside the organization/workplace, were identified.

*Internal organization-based barriers and facilitators.* Barriers within the organization included complex procedures to obtain and implement accommodations. To illustrate, the organization’s lack of flexibility in allocating resources and its lengthy bureaucratic processes were reported as barriers for obtaining accommodations [25,33,44,52]. The delay in providing necessary services or the lack of support systems in the workplace (e.g., clear guidelines) also created barriers to maintaining employment [33,52]. Unpreparedness of companies and organizations and the lack of awareness of existing policies and resources, as well as limited knowledge on how to implement those policies in their workplace, impeded the successful engagement in employment [53]. Specifically, knowledge on how to select and hire a person with disability, what type of accommodations to provide, and how to handle different situations was limited [30,32,34,47]. This issue was evident in organizations where accommodations were made based on the employers’ “recognition” and their “willingness/readiness” to provide services, or in organizations that determined the employee’s accommodation needs based on a strictly medical-oriented approach [33]. In such cases, the medical diagnosis rather than the employee’s level of function or needs informed the decision of providing accommodations. Limited funding to support awareness of employers and colleagues about disability [49] and insufficient recognition of various types of certificates or diplomas [40] further accentuated this barrier. Additionally, workplaces in which employees were not given constructive feedback, their abilities, skills and contribution were not recognized nor valued, and where they were not involved in the decision-making process, reduced opportunities to advance their careers [25,51,52].

Characteristics of the organization in terms of employment expectations (e.g., task demands, schedules) and availability of support services were reported as facilitators. Work settings that showed flexibility, especially in determining schedules and adapting job demands to the abilities of their employees, facilitated participation [38,44,54]. Flexible organizations that provided adequate accommodations (e.g., allocated more time, allowed work from home, provided breaks as needed, ensured consistent work routine) in a timely manner contributed to the employment of this population [25,33,50]. Those that provided individual-based support to their employees in work (e.g., communicated a change in medication to the employer; broke down or simplified tasks, set work goals, provided personal help to go to the bathroom) and non-work-related areas (e.g., helped adjusting to moving to a new residence) as well as guiding their employees on company policies, protocols and culture (e.g., taking time off for medical reasons), facilitated job sustainability [36,39,49,54]. Offering supervision and appropriate training on work demands and the social cues within the workplace, was another perceived facilitator [28,48,49,50,54]. The provision of ongoing support combined with clear job descriptions and expectations helped young adults maintain their jobs and progress in their careers [49]. Finally, organizations that promoted disability awareness and provided training for staff increased the likelihood of creating an engaging work environment for this population [25,49,50].

*External barriers and facilitators.* Factors external to the organization/workplace were also observed and involved both aspects of services and policies. In terms of access to employment supports and services, employees with disability expressed the need for more services to find employment as well as support in the workplace to maintain it. For example, young adults reported that employment services that helped with job applications, but did not assist in job searching that fitted their abilities, made finding employment difficult [40]. Additionally, scarcity of accessible employment and lack of professional support further limited their ability to enter the workforce [24,34,35,47,53]. Access to adult service agencies, disability employment services, job coaches, social workers and school staff, that provided training to employers and supported the employee on the job, facilitated transitioning to the workforce [44,48].

Policies addressing laws and regulations external to the organization, to support inclusion and workplace participation, also had an impact on successful employment as evident in a few studies. The availability of policies and their implementation in workplaces were mainly examined. Parents of young adults with developmental disabilities were concerned about the lack of macro-level policies supporting employment [26]. A study done in Namibia [43] revealed that inclusion policies for young adults with visual impairments were not effective in the workplace and were not implemented. Another study completed in both the United States and Norway highlighted that although some policies such as the Americans with Disability Act (ADA) recognized the rights of people with disability in the workplace and promoted “reasonable accommodations,” they were unclear about the extent and the range of assistance that should be provided. This resulted in the provision of inadequate assistance to the employee, impacting their ability to perform their jobs [33]. Different types of government programs had varying impacts on the access to employment of this population. For example, government wage subsidies were found to facilitate employment in some countries such as Sweden [33,36]. On the other hand, sheltered employment programs restricted the ability of the individual to acquire open and competitive employment in Australia [26]. Finally, young adults also expressed that the removal or reduction of government-based income benefits after acquiring well-paid employment prevented them from reaching their full potential at work [33,40,51].

#### 3.2.5. Other Contextual Factors

Contextual factors that did not fit any of the ICF environmental domains yet contributed to the employment of young adults with brain-based disabilities emerged and are grouped under personal factors. Examples include financial advantages, educational opportunities, and opportunities to participate in extracurricular activities and in the community (e.g., volunteering) [30,47]. Studies found that lack of previous work experience and lower levels of education contributed to fewer employment opportunities [31]. Similarly, Lindstrom et al. [46] and Lindsay et al. [34] concluded that higher levels of education led to broader qualified jobs with a higher salary within this population. Among the facilitators, Lindsay [37] showed that lower household income and fewer household members were associated with increased probability of having paid employment among individuals with cognitive or communication impairments. Young adults who benefitted from disability services and supports, and those who participated in the Co-op and internship programs offered through their high school and post-secondary schools were also found to have better employment opportunities [34,46].

## 4. Discussion

This scoping review revealed that all aspects of the environment as described by the ICF have an impact on workplace participation as a barrier and/or as a facilitator, expanding previous research conducted among those with ID [14] and ASD [13], to a broader range of brain-based disabilities. Specifically, a large body of evidence (77% of the studies) focused on the impact of services, systems and policies on both acquiring and maintaining a job. An emphasis was placed on the role of the organizations in creating an inclusive work environment, providing training for and promoting disability awareness of managers and staff, as well as embracing positive attitudes. As such, findings draw attention towards the developing of interventions that reduce the environmental barriers at the organizational level, identified in this review.

None of the studies examined the effectiveness of existing policies that specifically promote employment and workplace participation at the macro-level (i.e., provincial and national policies in the larger societal context). The few studies that mentioned “policies”, described the lack of awareness and at times, willingness to implement existing policies in the workplace. The same pattern was seen among older adults with disabilities who face work participation challenges due to either inadequate implementation of policies and regulation or the lack of it all together to support their work participation [55,56]. This further emphasizes the importance of implementing policies at early stages since that is when young people enter the work force. Furthermore, not only are there very few policies to promote the employment of this population but there are no clear guidelines and procedures on how to implement and reinforce them in the workplace. Future research can address this issue by developing adequate policies, proposing and testing effective ways to disseminate information on policies to stakeholders (e.g., managers, supervisors, employers and employees with and without disabilities) as well as finding adequate ways to implement them. This can be achieved by providing educational programs, as well as having clear procedures and processes in place to implement them.

Studies also demonstrated the positive impact of social support while shedding light on the detrimental effect of negative attitudes on workplace inclusion of this population. This finding supports the need for effective interventions by service providers and policymakers to improve attitudes in the work environment. This can be done through educational initiatives, increasing others’ knowledge about disability and inclusion as well as providing information on how to make successful accommodations in the workplace. Furthermore, findings highlight the use of assistive technology in enhancing work participation by facilitating the completion of certain work tasks and performance of responsibilities. With rapidly developing technological solutions, putting in place technology-based accommodations (applications, software) has become readily available [41], making the implementation of such accommodations more practical.

Several knowledge gaps were identified. Although the literature described a range of environmental barriers that impacted workplace participation, there is still little that is known on effective strategies to overcome these environmental barriers. Indeed, only seven studies (out of 31) described strategies used to facilitate work participation, without evaluating their impact. The available examples of actions that organizations can take, focused mainly on improving physical accommodations (e.g., providing assistive technology, giving extra time to complete tasks, creating an accessible environment), with little evidence on strategies to remove other important barriers like attitudinal (e.g., discrimination, pre-conceived ideas about disability), organizational (e.g., rigid task demands and schedules), and institutional (e.g., lack of training and support). In addition, the majority of the included studies were qualitative in nature. This can be complemented by quantitative studies using advanced statistical methods to systematically evaluate the environment and the workplace participation. Furthermore, most of the studies employed a cross-sectional design, with only two longitudinal studies, suggesting that available evidence is limited in claiming causal relationship between the environment and participation. Notably, while our approach to synthesize evidence according to the domains of the ICF appeared overall appropriate, only five studies (out of the 31) explicitly used the ICF as a guide. Finally, very few of the quantitative studies administered standardized, comprehensive and psychometrically sound measures to evaluate environmental factors that affect participation in the workplace.

The knowledge synthesized may guide employment-related service providers to identify specific environmental characteristics that are important, need to be evaluated, and are potential areas for intervention. Findings demonstrate that there is a strong promise in shifting focus toward the environment, rather than solely focusing on the skills of transition-aged individuals with brain- based disabilities. Interventions, programs and policies can target support and services at the institutional level (within a broader structural context such as social systems/community agencies) and organizational level (within the immediate workplace environment) as these factors were commonly identified as barriers/supports. This information can be used to develop or strengthen environment-based interventions, such as the Pathways and Resources for Engagement and Participation (PREP), proven effective in improving community participation among transition-aged young people by only changing aspects of their environment [57]. Policymakers can also draw on this knowledge to develop clear and specific guidelines to implement and reinforce policies in the work environment. Transition programs and services based in the community can also benefit from this knowledge by developing programs that address specific environmental barriers, faced by young individuals, and foster their inclusion in open and competitive employment.

A limitation of this study is that grey literature and articles not published in English were excluded, which may have resulted in important information being missed. Additionally, given that the aim of this review was to synthesize literature related to the impact of the environment on open and competitive employment, studies focusing on participation in sheltered employment were excluded. Thus, it is possible that information relevant to the environmental impact on employment participation was omitted. Typical to scoping reviews [21], no quality assessment of the included studies was conducted due to the large number of research designs and variety in methodological approaches of the included studies. Given that this topic is a newly studied area, the intent of this review was to synthesize all information available without parameters related to study quality. Thereby, no firm conclusions can be made about the effectiveness or the magnitude of the effect of the environment on work participation among young adults with brain-based disabilities.

## 5. Conclusions

Findings highlight the role of the environment in facilitating and/or hindering employment. Particularly, environmental factors at the organizational level and at the institutional level appear to be critical in fostering workplace participation in this population.

## Figures and Tables

**Table 1:** Search terms used.

Database	Environment [Combined Using OR]	Work Participation [Combined Using OR]	Disability [Combined Using OR]
1. OVID 2. MEDLINE 3. EMBASE 4. PsycINFO 5. PubMed 6. CINHAL	Physical environment Social environment Cultural environment Institutional environment Social support Relationship Attitude Accessibility Architectural accessibility Service Policy Built environment Environmental design Organizational climate	Employment Employment status Participation Involvement Engagement Workplace Work Job Vocational Part time job Productivity Volunteer Part-time work Labor market	Brain-based disabilities Cerebral palsy Brain hemorrhage Traumatic brain injury Cognitive impairment Epilepsy, post-traumatic epilepsy Hydrocephalus Meningitis, bacterial Meningitis, fungal Meningitis, viral Meningoencephalitis Child development disorders, Developmental disabilities Intellectual disability Learning disorders Motor skills disorders Tic disorders Global developmental delay Autism spectrum disorder Asperger syndrome Developmental coordination disorder Sensory integration disorder Sensory system disorder Disorder, Spina bifida Acquired brain injury

**Table 2:** The main findings of the individual articles (*n* = 31).

Author, Year, Country	Aim of the Study	Study Design	Population (Number, Age, Diagnosis)	Environmental Domains				Summary of Main Findings
				Products & Technology & Natural Environment	Support & Relationships	Attitudes	Services, Systems & Policies	
Foley et al. [24] Australia	To present parental descriptions of social participation of young adults with Down syndrome and to explore the levels of social participation with physical and social environment.	Quantitative—Cross-sectional study	*n =* 197 parents of youth Youth aged * 16–32 Down syndrome	✓	✓	✓		Facilitators: Positive attitudes of employers and colleagues Barriers: Negative attitudes of strangers Lack of support from friends Unavailability of jobs and public transport
Roessler et al. [25] USA	To demonstrate the application of a contextual assessment of job/person compatibility in four employed college graduates with TBI.	Qualitative—case study	*n =* 4 Aged 25–32 years TBI	✓		✓	✓	Facilitators: Flexibility to work from home Receiving positive reinforcement Employee assistance programs Allowing employees to contact doctors during work Altering work environment (lighting and temperature) as necessary Having clear employee responsibilities and creating goals for employees Barriers: Inadequate lighting, temperature and noise in the physical environment Fast work pace, large variety of duties, performing under pressure, limited feedback on performance, hostile coworkers, inflexible work schedules and unfitting sick/vacation leave policies. Insufficient time to work alone, little recognitio for the work completed, inadequate training from employer
Foley et al. [26] Australia	To describe the quality of life of families with a young adult with Down Syndrome, recently transitioned from school to post-school and influences of post-school day occupation and personal, environmental factors on family quality of life.	Quantitative —cross –sectional study	*n =* 150 families of young adults with Down Syndrome Aged * 16–30 years (mean = 22.9)	✓	✓		✓	Barriers: No suitable open employment jobs available Employees unable to apply for open jobs while working in sheltered employment Unreasonable travel distance Lack of parental support Policy and funding constraints Organizations providing inadequate support for employees with disabilities
Sung & Connor [27] USA	To investigate career behaviour, self-efficacy, goals, and contextual supports and barriers as predictors of choice actions and work participation among transition-age individuals with epilepsy.	Quantitative — cross-sectional design	*n =* 90 Aged 18–25 Epilepsy		✓			Facilitators: Work participation was positively associated (moderate) with supports (e.g., having a mentor to guide and encourage) and negatively correlated with barriers (e.g., lack of employer’s support) 58% of the variance in work participation was accounted for by environmental supports from family, friends and processionals (β = 0.238), self-efficacy with making career decisions (β = 0.221), and expectations related to the outcomes of working (β = 0.460)
Butterworth et al. [28] USA	To better understand the relationship between the characteristics of the workplace and the levels of support and social inclusion experienced by employees with a disability.	Qualitative—part of larger study	*n =* 8 young adults Aged * 17–22 Developmental disability		✓		✓	Facilitators: Managers showing personal interest in employees Strong sense of teamwork High levels of support (social opportunities, emphasis on shared job responsibilities, employee trainings for multiple jobs) Creating multiple in-depth relationships crossing over different life contexts
Barf et al. [29] Netherlands	To examine participation restrictions of a large group of young adults born with SB in relation to disease characteristics, activity limitations and perceived hindrances for participation.	Quantitative —cross-sectional study	*n =* 179 Aged * 16–25 years (mean = 21) SB	✓				Barriers: Building inaccessibility General costs Travel distance to workplace
Greenbaum [30] USA	To obtain information on employment and social status of college alumni (1980–1992) with learning disabilities.	Quantitative —cross-sectional study	*n =* 49 Mean age = 26 Learning disability	✓	✓	✓	✓	Facilitators: Family support College education and higher socioeconomic status Barriers: Only 20% of employees disclosed their diagnosis due to concerns about discrimination Employee’s lack of knowledge or willingness to exercise rights as outlined by the Rehabilitation Act of 1973 and the Americans with Disabilities Act of 1990
Honey et al. [31] Australia	To investigate the transitions between full-time, part-time and non-employment for young people with and without disabilities.	Retrospective—longitudinal study	n= 766 with disability, n=5008 without disability Aged * 15–29 Disability not specified		✓		✓	Barriers: Low social support and low education Current employment status was strongly linked to previous employment status
Toldrá & Santosb [32] Brazil	To identify facilitators and barriers faced by people with disabilities in the workforce.	Qualitative—Discourse of the collective subject matter method	*n =* 10 Aged 21–36, SCI, MD, CP, blindness, spinal amiotrophy, multiple arthrogiposis, congenital malformation	✓	✓	✓	✓	Facilitators: Building social relationships in the workplace Physically accessible environment Barriers: Prejudice Inadequate employee support by companies for workplace accommodations
Solstad & Schreuer [33] USA & Norway	To explore from a cross-national perspective, the complexities of workplace accommodation policies in action.	Qualitative study	*n =* 29 Age *: U.S.A: 22-39 (median 31) Norway: 24-43. (median:33) 2/3 CP, osteogenesis imperfecta, or SB.	✓			✓	Facilitators: Flexible or reduced work hours Accessibility to transit, physical work environment, assistive technology, and job coaching Ability to work from home Barriers: Timely transportation Lack of employer’s awareness about necessary accommodations Costs/length of implementing accommodations
Lindsay et al. [34] Holland and Canada	To explore the facilitators, barriers and experiences of employment and post-secondary education among youth and young adults with spina bifida; and their variations between youth and young adults with spina bifida, their parents and health care providers.	Qualitative—secondary analysis from larger study	*n =* 12 youths, 11 parents and 12 health care providers Aged 19–25 SB	✓	✓	✓	✓	Facilitators: Support from family and peers, participation in internships through school Having accommodations made through a disability service at the post-secondary educational level Barriers: Lack of supports and resources, limited options for accessible jobs, transportation, over-protective parents, stigma and discrimination, employer stereotypes, lack of professional support to find employment, and work tasks unfit with the employee’s physical skills
Sherer et al. [35] USA	To explore the prognostic value of self-reported traits, problems, strengths and environmental barriers or facilitators for participation outcomes in persons with traumatic brain injury (TBI).	Systematic review	*n =* 63 articles >17 years old TBI	✓	✓		✓	Facilitators: Access to transportation Services and social interaction
Törnbom et al. [36] Sweden	To compare work participation in 2009 with 1997 in individuals with cerebral palsy and spina bifida.	Longitudinal —descriptive study	*n =* 30 Mean age 24 CP and SB	✓			✓	Facilitators: Access to personal assistance Adequate transportation Implementing necessary accommodations Continuing education Wage subsidies to employers Barriers: 29% of employees used transportation for people with disabilities in 1997 compared to 50% in 2009. This type of transportation was criticized because of frequent late arrivals and long travel times
Lindsay [37] Canada	To explore the characteristics associated with disabled youth who are employed and the types of employment they are engaged in.	Retrospective—cross-sectional study	*n =* 5234 Aged * 15–24 years old mobility, hearing, vision, communication, cognitive impairment	✓	✓		✓	Facilitators: Access to vehicle Being in urban setting Fewer people in a household with a low total household income
De Beer et al. [38] Netherlands	To determine facilitators and barriers associated with participation in work of individuals with developmental disabilities, classified according to the dimensions of the ICF.	Systematic review	*n =* 256 Mean age = 33 Developmental dyslexia and/or learning disability	✓	✓	✓	✓	Facilitators: Support from employer and colleagues Access to assistive technology Barriers: Support and relationships, attitudes of co-workers, working conditions, legal services, systems and policies, social security service systems, policies, SES and education level.
Ripat, & Woodgate [39] Canada	To present experiences and use of assistive technology (AT) from young adults in supporting their productivity.	Qualitative—grounded theory and participatory research study	*n =* 20 Aged * 17–35 SCI, CP, SB, MS, non-verbal disorders, dyslexia, visual impairment, Usher’s and Ehlers–Danlos Syndrome	✓		✓	✓	Facilitators: Access to AT Active engagement in accommodation duties Barriers: AT was sometimes seen as unnecessary by co-workers and was viewed as a privilege. Cost of AT
Darrah et al. [40] Canada	To understand the contribution of educational, employment, transportation and assured income service programs to the successful transition of young adults with motor disabilities to adulthood.	Qualitative study	*n =* 76 Aged 20–30 CP and SB	✓			✓	Barriers: Concerns with having reduced income benefit, lack of accessible transportation, limited post-secondary training opportunities, lack of employment accommodations, and a lack of services available to assist with finding a job.
Morash-Macneil et al. [41] USA	To investigate the efficacy of assistive technology (AT) in improving the ability to complete work tasks independently and efficiently for individuals with intellectual disabilities.	Systematic review	n=29 Aged *: 15–24 ID	✓				Facilitators: Appropriate assistive technology such as portable electronic devices resulted in improved employment skills like task completion, time management and increased productivity
Holwerda et al. [42] Netherlands	To investigate factors that predict work participation, finding and maintaining employment of young adults with ASD and as ADD.	Longitudinal - cohort study	*n =* 563 Aged * 15–27 (mean = 19.4) ASD and ADHD		✓	✓		Facilitators: Positive attitude and support from parents and others at work Barriers: High parental support: overprotective parents might prevent children from finding employment
Tobias & Mukhopadhyay [43] Namibia	To identify the social experiences of individuals with a visual impairment in rural Namibia and to provide suggestions on how to include them in the community.	Qualitative study	*n =* 9 Aged 30 to 90—information was extracted from 3 participants who were in their 30s Vision impairment		✓	✓	✓	Barriers: Lack of social and family support restricted access to education The abilities of participants with vision impairment were undermined due to being viewed as dependent. Policies promoting the employment of people with visual impairments were not enacted.
Hagner et al. [44] USA	To clarify the current implemented strategies to facilitate the involvement of natural support resources in the employment process.	Qualitative study	*n =* 33 vocational specialists/staff Age of participants not specified as study was completed from perspective of vocational support specialists		✓		✓	Facilitators: Support from family and friends, social interaction among co-workers, and inclusion of company personnel in the training of an employee with a disability Barriers: Low family involvement: unwillingness to assist in job searching due to lack of time, being overprotective, embarrassment related the youth’s disability or not believing that the youth could succeed in a job Lack of flexibility of company resources and resentment or discrimination toward individuals with disabilities
Petner-Arrey et al. [45] Canada	To better understand the experiences of people with intellectual or development disability (IDD) gaining and keeping productivity roles	Qualitative—grounded theory	*n =* 74 (13 persons with IDD, 21 caregivers, 40 pairs of caregivers and people with IDD Aged * 21–54 (mean = 27)		✓			Facilitators: Parents and social networks facilitated acquiring and sustaining employment providing on the job assistance, helping employees to understand job expectations and providing advocate support
Lindstrom et al. [46] USA	To examine the career development process and postschool employment outcomes for a sample of individuals with disabilities.	Qualitative—case study	*n =* 8 Aged 25–28 learning & emotional disability, orthopedic impairment		✓			Facilitators: Previous work experience Positive interactions with colleagues Completion of higher education and career supports in high school
Lindsay et al. [47] Canada	To explore the extent to which youths with physical disabilities encounter barriers to employment compared to their typically developing peers.	Qualitative—part of larger multi-method study	*n =* 31 youth (16 typ. Dev. And 15 with disability); 9 youth employers, 10 job counselors Aged * 16–19 CP, MD, myoltubularmyopathy, central core myopathy, Guillianbarre, scoliosis	✓	✓	✓	✓	Facilitators: Peer influence helped motivate youth with disabilities to seek out employment Financial incentive for employers to hire employees with disabilities Barriers: Parental overprotection Inadequate development of social and communication skills needed for the workplace Inaccessible environments and challenges with advocating for accommodations Concerns related to disclosing diagnosis, perceived disadvantages as a result of employer stereotypes and potential loss of disability benefits Employers’ lack of knowledge on how to adapt the environment, training procedures and tasks to support employees with disabilities Lack of funding to support employers’ awareness of disability
Reid & Bray [48] New Zealand	To present opinions of workers, supporters and employers and to offer strategies for greater employment rates and better-informed decisions by education, training and support agencies.	Qualitative study	*n =* 17 workers, 3 employers, 7 support people, 2 experts on employment Mean age early 30s (range 24–50) ID		✓		✓	Facilitators: Engaging in social activities, having flexible work hours, access to services to assist with finding and maintaining employment
Scott et al. [49] Australia	To present and contrast the viewpoints of adults with ASD and employers for successful employment and to explore how these viewpoints impact the process of employment.	Qualitative—Q method	*n =* 40 employees *n =* 35 employers Employee: Mean age: 29.1 Median: 26 Employer: Mean age: 44.6 Median: 44 ASD	✓	✓	✓	✓	Facilitators: Having an inclusive work environment, continued support from an employment support worker after hiring, approachable manager, and investing in inclusion Workplaces that valued, encouraged and supported the employee
Li EPY [50] China	To look critically at the competitive employment experiences of people with intellectual disability and at their perception of social barriers that could affect their ambition to get a job in the community.	Qualitative study	*n =* 18 Aged * 22–43 (mean = 28.7) Mild ID		✓	✓	✓	Facilitators: Positive attitudes and support from employers and colleagues Assistance from professionals for employment, disability education for public and employers, training programs to support the development of work and social skills Barriers: Stress of the interview and negative attitudes of the employer Workplace discrimination, poor relationships with co-workers and employer
Roessler et al. [51] USA	To determine whether the nature and scope of workplace discrimination is different for youths with epilepsy as compared to other types of disabilities.	Quantitative —comparison analysis	Epilepsy: *n =* 555; General Disability: *n =* 12,663 allegations Aged 18–25 Epilepsy			✓	✓	Barriers: Job retention was impacted by allegations of discrimination, stereotypes about epilepsy, and frequently being hired into less secure entry level jobs Unlawful discharge was higher in youths with epilepsy compared to the general disability grouping
Wilson-Kovacs et al. [52] United Kingdom	To present barriers, problems and potential solutions to challenges that members of marginalized groups encounter in the workplace.	Qualitative study	*n =* 14 Data presented for those 35 years old Polio, hearing loss, MS, dyslexia			✓	✓	Barriers: Lack of feedback provision and inclusion in decision making, perceptions of employee ability, discrimination, lack of necessary accommodations to support integration into workplace culture
Lieketseng & Lorenzo [53] South Africa	To describe the capacity of service providers in facilitating the participation of disabled youth in economic development opportunities	Qualitative—case study	*n =* 5 disabled youth, 4 family members and 6 service providers Age only specified as youth Intellectual or sensory impairment			✓	✓	Facilitators: Disability grants for young adults with disability who want to start their own business Barriers: Lack of knowledge about the need for inclusion and how to support it, attitudes, stereotypes about disabled youths’ participation in the workplace and lack of enactment of inclusion policies Disability grants for young adults with disability limit work opportunities
Hagner & Cooney [54] USA	To locate individuals with autism who were successfully employed at jobs in the community and to identify the factors that contributed to their success.	Qualitative study	*n =* 14 Aged * 23–36 ASD		✓		✓	Facilitators: Job modifications such as maintaining a consistent schedule, flexibility in job training, completing the same set of work duties and providing a checklist of tasks that need to be completed Supervisors providing information about social cues, rules and direct instructions for work tasks For employees with ASD: coworkers initiating conversations and providing feedback regarding social conventions
			**Total:**	**17 (55%)**	**21(68%)**	**15 (48%)**	**24 (77%)**	

ID: Intellectual disability, SB: Spina bifida, SCI: Spinal cord injury, CP: Cerebral palsy, MS: Multiple sclerosis, TBI: Traumatic brain injury, MD: Muscular Dystrophy, ASD: Asperger Spectrum Disorder, ADHD: Attention deficit hyperactivity disorder. * Age: Studies with participants below 18 and above 35 years old are included because the mean age of participants in the study lies within 18–35 years old and/or they provide results for a subset of the participants within the range 18–35 years old.

**Table 3:** Examples of environmental barriers and facilitators across the ICF domains.

Domains	Facilitators	Barriers
**Products & technology/Natural environment**	Physical alterations of the building and/or equipment, accessible path, ramp, door handle, open and lock door system, accessible bathroom, separate office, and adjustable desk [33] Specialized assistive technology such as voice recognition software, special mouse, or computerized phone [33,38,39,41] Living in urban cities [37]	Transportation: lack of access, long distance [29,33,36,40] Difficulty navigating public transport [34] Inadequate lighting and temperature in the work setting [25]
**Support & relationships**	Support from the employer [38] Support from colleagues (e.g., proofread work) [30] Support from family and friends to connect young adult with disability to work opportunities [45] Support from parents (emotional, help with transportation, finding employment, teaching independence skills) [30,34,44] Positive interactions with colleagues at work (e.g., lunch, breaks) and during non-work related activities [28,44,46] Receiving information from colleagues about etiquette and dress code when participating in work-related social conventions [54] Approachable managers who promote fair workplace setting [28,39,49]	Poor relationships with employers and co-workers [50] Overprotective parents [34] Lack of support from parents in job search [43,44]
**Attitudes**	Positive attitude from colleagues towards people with disability [50]	Employer who does not believe in the abilities of a person with disability [30,32,52] Employers’ attitude, misperceptions and stereotypes [50,51] Discrimination [30,34,51,52] Negative reaction upon disclosure of condition [38] Being alienated by colleagues and co-workers if using assistive technology [39] Employer’s belief that employing people with disability is costly due to their needs for accommodations [52]
**Services, systems & policies**	Settings that promote inclusion, fair workplace and high levels of interactions and support [49] Flexible work demands (schedules, workload) [30] Workplaces that value and recognize employee’s skills and contributions [49] Availability of support services and training programs for employers as well as employees [44] Receiving assistance from professionals to find and maintain job [50] Ongoing support from disability employment service providers when making workplace adjustments [49] Policies that promote reasonable accommodations based on the employee’s needs [33] Wage subsidies in some countries such as Sweden [36] Opportunities to continuing education [36]	Unpreparedness and lack of knowledge from the company on how to accommodate a person with disability [30,32,47] Lack of available jobs [26] Lack of knowledge regarding policies and available services [30] Lack of clear policy implementation guides for workplaces [47,53] Limited reinforcement of existing policies [43,53] Certificates or diplomas that are not being recognized by workplaces [40] Eligibility for accommodations is based solely on medical diagnosis rather than employee’s needs or functional levels [33] Lack of professional support in job search [47] Slow delivery of services [44] Inflexible work schedule [25]
**Other contextual factors**	Higher family SES [30] Higher level of education [46,47] Fewer number of people in the household and lower SES [37] Participation in internship and co-op programs [47]	Few opportunities to participate in extracurricular or social activities [47] Lack of opportunities to volunteer [47] Low education levels [31]
